# Factors Affecting the Welfare of Calves in Auction Markets

**DOI:** 10.3390/ani9060333

**Published:** 2019-06-08

**Authors:** Viviana M. Bravo, Toby G. Knowles, Carmen Gallo

**Affiliations:** 1 Programa Doctorado en Ciencias Veterinarias, Escuela de Graduados, Facultad de Ciencias Veterinarias, Universidad Austral de Chile, Casilla 567, Valdivia 5090000, Chile; 2 School of Veterinary Science, University of Bristol, Langford, Bristol BS40 5DU, UK; toby.knowles@bristol.ac.uk; 3 Instituto de Ciencia Animal and OIE Collaborating Centre for Animal Welfare and Livestock Production Systems, Facultad de Ciencias Veterinarias, Universidad Austral de Chile, Casilla 567, Valdivia 5090000, Chile; cgallo@uach.cl

**Keywords:** calves, auction market, movement, penning, behavior, handling, facilities, welfare

## Abstract

**Simple Summary:**

Marketing is described as inherently stressful for cattle. When animals are sold through auction markets, travel time and fasting may be prolonged, and the chance of being mixed with unfamiliar animals is increased. Within Chile, 37% of auctioned cattle are “calves”. To assess factors that may be affecting the welfare of calves during handling and penning in markets, twelve markets were visited. Behavioral indicators of welfare during movement and penning were observed. Handling and facilities were also evaluated. We determined variables associated with differences in behavioral indicators of welfare between groups of calves. An increase in group size, number of blows/hits and other negative interactions, presence of aggressive/rough driving, slippery floor, and inadequate lighting were associated with differences in welfare indices during movement. During penning, not mixing calves from different farms was associated with an increase in positive and a decrease in negative behavioral indicators. Increased stocking density was associated with decreased positive indicators. The increased numbers of daily auctioned cattle, passage of time, and calves not mixed with incompatible animals were associated with a decrease in negative indicators and the presence of males was associated with an increase. Variables associated with negative welfare indicators were mainly linked with handling of calves.

**Abstract:**

Marketing cattle through auctions increases marketing time, exposing animals to more stressful events. Within Chile, 37% of auctioned cattle are “calves”. To assess factors that may be affecting the welfare of calves during movement and penning, twelve markets were visited to evaluate behavioral indicators of welfare, handling, and facilities. Behavioral indicators during movement were classified into movement and other behaviors, and indicators during penning were classified into positive or negative behavioral welfare indicators. For each group of calves, an index was calculated based on a proportion of observed behavioral indicators. Statistical models were built to identify variables associated with changes in these indices. Presence of inappropriate driving, inadequate lighting, and slippery floor was associated with a movement index increase (poorer welfare). Negative tactile interactions were associated with an increase, and group size was associated with a decrease in movement index and other behavior indices. During penning, not mixing animals from different sources was associated with an increase and stocking density with a decrease in positive welfare index. Number of auctioned cattle, observation number, and not mixing with incompatible and/or with calves from different sources were factors associated with a decrease in negative welfare index, and the presence of males was associated with an increase. Behavioral welfare indicators were mostly associated with handling.

## 1. Introduction

Cattle undergo a live marketing process at least one time in their productive lives; they may be sold to another farm or sent to the auction market or the abattoir according to their stage of production. Marketing is described as inherently stressful, as it requires that animals are removed from their environment, transported, and housed in unfamiliar surroundings [1]. Selling cattle through auction markets especially increases the travel time, as travel both to and from the site is required; fasting may be prolonged, and there is an increased chance of being mixed with unfamiliar animals [1,2].

In Chile, some beef cattle producers complete the entire production cycle themselves, from breeding to finishing, whilst others sell their calves after weaning for further fattening to specialist finishers [3]. Within Chile, for beef calves (animals under nine months of age), weaning stress is an additional stressor linked with marketing, as it is often accomplished simply by transporting the animals away from their dam at the time of sale. Phillips [4] describes weaning and transport as the two major stressful events in the lives of calves. Further compounding the cumulative stress, these animals are often transported to auction market rather than directly to a finisher. During 2018, 940,396 cattle were auctioned in markets in Chile, 37% of which were calves [5]. Selling animals through auction markets increases handling (double loading and unloading together with movement through the market and penning), consequently exposing them to a greater number of interactions with humans. A study conducted in Chilean auction markets evaluating the quality of human-animal interactions (HAI) involving stockpersons found that most of the staff observed (65%) had negative HAIs [6].

Several studies related to pre-slaughter handling within Chile [7,8] and in other countries [9,10,11,12,13] have shown that the welfare of cattle sold through auction market is poorer compared with animals delivered directly to the abattoir. To be able to improve the welfare of the animals moving through markets, it is necessary to determine which are the factors having negative effects on their welfare. To assess the welfare of the animals, animal-based indicators are considered the most valid approach, since it requires the assessment of the animals themselves [14]. Some of these indicators have been reported previously in the assessment of cattle welfare during handling and penning under commercial conditions [11,15,16,17]. The aim of the present study was to assess possible factors that may be affecting the welfare of beef calves during movement (on the hoof) and penning in auction markets.

## 2. Materials and Methods

Twelve auction markets in the southern regions of Chile were visited twice. In order to evaluate possible seasonal differences, the first visit was performed during spring months and the second in winter. Markets were selected by availability (working all year round, at least fortnightly) and based on the number of calves observed during unloading (≥50 calves) in previous visits to all southern markets (*n* = 21). The Bioethics Committee “Use of animals in research” of the Universidad Austral de Chile approved the present study (Application N 325/2018).

The passage of the animals through the market was assessed in five stages, which are defined in Table 1. In each stage, a professional trained in animal behavior and welfare observed groups of calves between 150 and 250 kg (approximate weight at weaning) to measure behavioral indicators of welfare during handling and penning. Features related with facilities and handling and/or management were also assessed independently in each group of calves observed at each market, thus animals were handled under comparable but not identical conditions at the different markets. To maintain consistency, the distinct stages were always assessed by the same observer throughout the study.

Behavioral welfare indicators, handling, and facilities at the market were evaluated in each stage following a printed guide based on the scientific literature, the current animal protection Chilean law, and pilot visits to markets [13,15,18,19]. The number of daily auctioned cattle was also recorded as an indicator of throughput of the market.

### 2.1. Welfare Indicators of the Calves during Movement through the Market

To assess the calves’ welfare in the stages when there was movement of calves (all stages in Table 1 except penning), the following indicators were evaluated:

Behavior of calves: These were quantified in each observed group of animals in accordance with definitions provided by Maria et al. [15]—slip, fall, balk, turn, jump, vocalization, mount, aggression, and defecation/urination. Start and end time and the number of individuals per group were also recorded. This included a count of other species involved as necessary, as on occasion, mixed species groups could arrive at market.

The following variables relating to handlers and facilities were also evaluated as possible features that could affect and/or disrupt the normal passage of the animals being driven. Each feature was evaluated in each group of calves in each stage independently.

Handler: The device was used for appropriate driving (yes/no). Driving was described as appropriate when conducted quietly and without unnecessary noise, harassment, or force. Handler-behavior was measured using a count of negative tactile interactions with the calves (blows/hits and pokes with the device used to drive the animals, kicks and flex/pull of the tail).

Facilities: Floor type (slip-proof/slippery), obstacles/distractors in the path of the calves (presence or absence), and lighting (appropriate/inappropriate) were recorded for each stage per group. Lighting was described as inappropriate/inadequate when shadows or darkened places/raceways interrupted the movement of the calves.

Additional measures: During the unloading/loading stages, the features evaluated included who performed the procedure (truck driver/market staff/both); if both doors of the truck were opened before unloading or loading (yes/no); if the truck was parked correctly against the ramp, avoiding gaps and sideward deviation (yes/no); if the truck was parked level with unloading/loading facilities (<20 cm high between the truck and the ramp) (yes/no); and the slope of the ramp used [19]. Ramp slopes were obtained by simply measuring the height and the length of the ramp using a tape measure. It was also registered whether the handler was riding a horse during grading (yes/no) and the number of rings operating simultaneously during auction (one/two).

### 2.2. Welfare Indicators of the Calves during Penning

To evaluate penning, four pens of calves were selected at random at each market. Calves in pens were observed five times (observation number) for 5 min every 30 min. Before starting the observations, health status of the calves was visually inspected. In each pen, the number of calves was recorded. Pen groups were described according to sex (females, males, and mixed pens of males with neutered males) and breed type of the calves (meat, dairy, mixed).

Behavior of calves: The number of animals lying down (animal lying on the floor in lateral or sternal recumbency), ruminating (visible chewing and belching, not associated with food intake), mounting (animal mounts another animal), and performing aggressive bouts (antagonistic behavior between animals) was quantified. 

Handling: Mixing calves from different sources (yes/no) and if calves were mixed with incompatible animals (yes/no) were factors recorded. Animals were described as incompatible when they corresponded to another species, e.g., pigs, sheep, and horses, or to a different category of cattle, e.g., heifers, bulls, steers, and cows. Stocking density was calculated based on an average of weights recorded during weighing at the auction (kg/m^2^). Pen area (m^2^) was obtained by measuring the width and the length of the pen using a tape measure.

Facilities: Pens were assessed based on floor type (slip-proof/slippery), protection afforded against environmental conditions (presence or absence), and presence/absence of sharp edges that could damage the calves.

Environmental variables: Air temperature (°C) and relative humidity (%) were recorded in situ during each observation using a thermohydrometer (Elitech, model RC-4HC).

### 2.3. Data Analysis

Statistical models were built to identify handling, facilities, and environmental variables that were associated with the differences in recorded behavioral indicators of the calves during handling and penning.

Behavioral indicators during handling were counted and classified as: movement related behaviors (slip, falls, balks, turn, and jumps) and other behaviors (vocalizations, mounts, aggressions, and defecation/urination). For each type of event, a proportion was calculated based on the number of observed events per group divided by the number of calves per group to give an event index. Behavioral indicators during penning were analyzed, classifying events as positive (lying down, ruminating) or negative welfare indicators (mounts, aggressions). A proportion based on the sum of number of events per pen divided by the number of calves per group was calculated to give an index per category of events.

Descriptive analysis was performed using IBM SPSS Statistics version 25 to identify which variables were associated with the differences between groups of calves (as the statistical unit) in the average movement related behavior index (MI) and the other behaviors index (OI) and during penning through the positive welfare index (PWI) or the negative welfare index (NWI). Multilevel model analyses were performed using the software MLwiN 3.03 [20]. A multilevel modeling approach was employed to account for the clustering as a random effect and the repeated measurement structure of the data, e.g., groups within auction market and repeated visits to market. Predictor variables were retained within models at α ≤ 0.05. A graphical inspection of the residuals was made to check for normality of errors and homogeneity of variance.

## 3. Results

### 3.1. Welfare Indicators of the Calves during Movement through the Market

The first recorded handling observation started at 08:42 and the last one at 20:19. A total of 800 groups of calves were observed, which corresponded to 7258 calves, 649 other categories of cattle, 12 sheep, 11 pigs, and three horses. Table 2 shows the overall observations and minimum, maximum, and mean (in standard deviation) values of movement and other event indices obtained in each stage per season for the groups of animals. Index values show the proportion of events per calf recorded in each group, thus higher values represent more events and, consequently, generally poorer welfare. However, it needs to be noted that not all calves necessarily underwent an event type, as it could be that all the counts of an event came from only one calf or from a subset of the group.

The most frequent type of device used to drive a group of calves were wooden sticks (26%) followed by plastic pipes (25%); sharpened ends to poke animals in both wooden sticks and plastic pipes were observed. Also observed was the presence of nails deliberately fitted in wooden sticks at two markets used to poke the animals. The use of paddles (15.8%) and flappers (8.7%) was also seen, but these were only ever observed in use in and around the auction ring. The use of electric prods (0.6%), internal wooden struts from trucks (0.6%), and flags used correctly (0.3%) and incorrectly (2.3%) was also seen. The incorrect use of flag was associated with poking and hitting calves using the device. The use of a mixture of these devices and the use of no device were also recorded (15.9%).

The highest average MI was obtained during loading in winter (0.61) followed by grading in spring (0.60); the lowest index was found during unloading and was similar in spring (0.35) as in winter (0.33). The lowest OI was obtained during loading in spring (0.09) and winter (0.02). The highest mean OI was found in the auction ring during spring (0.46) and winter (0.54). In all classified events (except movement scored during grading in winter), a minimum value of 0.00 was recorded for at least one group. Therefore, it was possible for some groups to record no negative welfare events at all.

The most parsimonious multilevel models obtained describing the relation between MI and OI and the explanatory variables related to handlers and facilities are shown in Table 3. The models show that there was an average increase of 0.164 in MI for every extra unit increase in handler tactile interactions. When driving the animals was carried out with unnecessary noise, harassment, or force, MI increased by 0.103. An increased number of animals per group had an inverse effect on MI; with the group sizes between one to 60 individuals, for every extra animal added, MI decreased by 0.012. A slippery floor was associated with an increase of 0.261 in MI over a slip-proof floor, and inappropriate lighting was associated with an increase of 1.242 in MI compared with calves handled with proper lighting.

The predictive variables associated with significant changes in OI were handler tactile interactions and group size. For every extra point of handler tactile interaction, OI increased by 0.024, and for every extra animal in the group handled, there was, on average, a decrease of 0.016 in OI. 

When market and season were also added into the MI and the OI models as a predictive variable, the parameter estimates shown in Table 3 were not materially different. Similar results were found when the evaluation stage was added as a predictive variable. The number of cattle auctioned daily, the type of device used to move animals, and the presence of obstacles and distractors in the path of the animals were not significantly associated with either MI or OI. The mean, the minimum, and the maximum of the continuous predictor variables included in the models are shown in Table 4.

When the relationship between the MI and the observed features were analyzed exclusively during loading and unloading stages, sample size decreased, modifying the significance of some variables. If driving the calves was not carried out quietly by handlers, the significance became *p* = 0.839, and the presence of a slippery floor became *p* = 0.608, therefore no longer significant within these stages alone. The incorrect leveling of the truck with the ramp was the only explanatory variable exclusive to these stages that was significant (*p* = 0.005). An inappropriate leveling increased MI by 0.238 during unloading and loading. When OI was analyzed exclusively during loading and unloading, the variable “group size” lost significance (*p* = 0.50), and none of the explanatory variables evaluated within these stages alone were significant. Neither “If the handler was riding a horse during grading” nor the “presence of one or two auctions ring working simultaneously” had a significant association with MI or OI.

### 3.2. Welfare Indicators of the Calves during Penning

A total of 475 observations (2138 calves in 95 pens) were obtained, of which just 413 were valid, as at some observation occasions, calves were not present in a pen because they were in the auction ring or had already been loaded and transported away. All pen observations were made between 01:25 to 05:54. All observed calves were classified as physically fit to be marketed. Of the total of observed pens, 39% were only calves from beef breeds, 12% contained only dairy calves, and 49% with a mix of both. The average air temperature recorded in spring was 27.6° (range 19–37°) and 15.6° during winter (9.3–22.4°). The recorded mean relative humidity during spring was 40.9% (26–79%) and 67.1% in winter (48.9–85.3%). PWI and NWI recorded per season are shown in Table 5. In both seasons, the value of NWI was higher than PWI. Both PWI and NWI were higher during spring compared with winter (Table 5).

In exploring the relation between PWI and NWI and the explanatory variables, the models that are presented in Table 6 were obtained.

Not mixing groups from different sources increased PWI by 0.092 when compared with pens where calves were mixed. In the model, animal weight was available for a subset of the data and, consequently, a calculation of stocking density was available for just these pens (245 observations). The results for this variable were significant (*p* = 0.014) but are reported separately, as the reduced sample size reduced the power of the final model. For every 1 kg/m^2^ of increase in stocking density as measured at the time of the pen assessment across a range from 16.61 to 427.8 kg/m^2^, there was a 0.00048 decrease in PWI. The mean, the minimum, and the maximum of the continuous predictor variables included in the penning models are shown in Table 7. 

In relation to NWI, it was found that an increase in the number of daily auctioned cattle per market by one animal was associated with an NWI decrease of 0.0002. An association between NWI and the sequence number of an observation was found—for each later observation (each additional 30 min), the index decreased by 0.028, showing a reduction of the behavioral indicators of poorer welfare over time.

Not mixing groups of unfamiliar animals—both incompatible and from different sources—decreased NWI by 0.530 and 0.365, respectively, when compared with pens where calves were mixed. The presence of male calves in the pens was also a significant predictor variable for NWI. The presence of males increased NWI by 0.455 over pens with only females.

## 4. Discussion

### 4.1. Welfare Indicators of Calves during Movement

The objective of this study was to assess the factors that could be affecting the welfare of calves during movement and penning in auction markets. In short, blows and hits, noisy and aggressive driving, slippery flooring, and inappropriate lighting were all identified as reducing welfare in terms of the behavioral measures seen and the indices used, whilst larger group size tended to be associated with better welfare.

We found the lowest values (better welfare) of the movement index during unloading compared with other stages of marketing. However, in both seasons, the other behaviors index was lower than the movement index, showing a greater prevalence of adverse movement behaviors such as slips, falls, balks, turns, and jumps compared with other behaviors such as vocalizations, defecation/urinations, aggressions, and mounts during handling. Gregory et al. [12] also reported a low frequency of handling problems with cattle during unloading at auction markets in the United Kingdom (UK). They describe that the main problems were the occurrence of slips and falls at this stage. The highest movement index was obtained during loading in winter followed by grading in spring. Loading has been described as more difficult and stressful than unloading and consequently has more adverse effects on the animals’ welfare [12,14]. The high prevalence of refusals and balking during loading in markets also leads to inappropriate handling [12].

The highest mean values of the other behaviors index were obtained in the auction ring during spring and winter. Observers reported that a large number of these behaviors were associated with some features of this stage. Most of the time, the calves were auctioned alone or in small groups, and they were sorted and then driven into an auction ring, where they were suddenly exposed to many people, had a close interaction with the handler and other humans around, and were introduced to a noisy environment. Novelty is a major stressor to cattle, especially when they are suddenly confronted with it [20]. The sudden, unexpected appearance of a human incites fearful behavior in cattle [21]. Behaviors such as defecation, urination, and vocalization (all included in the other behaviors index) are widely considered behavioral indicators of acute stress or fear in cattle [22,23,24].

Gregory et al. [12] mention that slippery floors were one of the main contributors to slips and falls during grading. We found that, generally, calves were handled on non-slippery floors, but the lack of appropriate floor type was a predictor variable for an increase in the movement index. Often when the floor was described as slippery, we noticed that the surface was slippery due to wear through heavy use over the years. In one of the newest markets, we observed a lack of appropriate floor type because all the surfaces were of smooth concrete. In the same market, it was found that a great proportion of ramps had slopes steeper than that recommended and required by law and included the steepest recorded in the study. This demonstrates a current lack of knowledge within the industry regarding the design of facilities and an ignorance of systems appropriate to the livestock and the requirements of the current Chilean legislation [18].

There was a relatively low incidence of the presence of inappropriate lighting, but it was sufficient for it to be a significant predictor of an increase in movement index. The presence of shadows in the raceways leads animals to balk, because they will often stop and put their heads down to inspect the contrast [20]. Without the required time, calves are not able to distinguish the nature of this “obstacle”, and they can often be seen to jump over it. As with other grazing and herding animals that are prey species, fear motivates cattle to be alert to the necessity to escape from predators [20]. We found that driving calves noisily and with unnecessary harassment increased the movement index. Handling calm cattle is easier than handling agitated and fearful cattle [20]. In order to avoid escalating handling problems, cattle should be handled calmly and appropriately.

The group size and the “handler negative tactile interactions” were predictive variables in both the movement index and the other behaviors index models. On average, an increase of one calf in the group size led to a decrease in both indices, consequently improving animal welfare. Bigger groups showed fewer problems during driving through raceways. As herding animals, an animal separated from its group soon becomes agitated and consequently more difficult to handle [20]. Moreover, hitting is a painful stimulus that obviously has negative consequences for the animals [21]. Additionally, it is forbidden under the current Chilean regulations [17,18]. For cattle (and pigs), aversive actions by humans that will increase fear include hits, slaps, and kicks by the stockpersons, whereas pats, strokes, and peoples’ hand resting on the animal will tend to decrease an animal’s fear of humans [21]. It has been shown that hits, pokes, and shouts lead to high reactivity and antagonistic behaviors in the animals handled in auction markets [25]. De Vries [5] mentioned that training focusing on the working groups of stockpersons is likely to improve human–animal relationships at the livestock market.

### 4.2. Welfare Indicators of Calves during Penning

During penning, we found that not mixing animals from different sources was associated with an increase in the positive welfare index and a decrease in the negative welfare index. Stocking density was associated with a decrease in the positive welfare index. Increased number of auctioned cattle, the passage of time measured as the observation number, and not mixing with incompatible and/or with calves from different sources were associated with a decrease in the negative welfare index. The presence of males increased the negative welfare index.

We found higher values of the negative welfare index over the positive welfare indices in both seasons. Also, both indices tended to be higher during spring compared with winter (Table 5). Mixing calves from different sources was frequently observed. De Vries [5] also reported that mixing unfamiliar animals is a common practice in Chilean auction markets, and that when this occurred, animals showed agonistic behaviors and appeared excited, showing behaviors such as vocalization, butting, mounting, and defecation. Mounting is a social behavior likely related to dominance and appears to be a symptom of a social environment to which the animal has no behavioral means to adapt to because there is little space or a large group size, group instability, or both [26]. It is known that mixing groups of unfamiliar animals at any stage during marketing leads to an increase in antagonistic behaviors [2]. Keeping animals in their original rearing groups to prevent problems is obvious but often difficult to achieve in practice [1]. At mixing, both aggression and mounting increased, and the two declined at the same rate with time [27,28]. The predictive variable “observation number”, representing the time animals remained penned, was significant in our negative welfare index model, demonstrating that behaviors such as mounting and aggression—both included in our negative welfare index—decreased over time.

We found an effect of the number of auctioned cattle (representing the size of an auction market). The increase in the number of daily auctioned cattle decreased the negative welfare index, improving the indicator. The minimum, the maximum, and the mean number of daily auctioned cattle found are shown in Table 7. Gregory [12], at markets in the UK, also found fewer problems related to handling in large auction markets (>300 animals in a market) compared with those of a medium or a small size (<300). However, De Vries [6] mentioned that the number of agonistic behaviors such as vocalization, butting, mounting, and defecation seemed to be lower in quiet markets compared with the busy ones. The decrease in behaviors indicative of negative welfare found within markets auctioning a higher number of cattle could possibly be explained by a greater number of available pens leading to less mixing of unfamiliar calves (incompatible or/and from different sources). It is possible that workers attempt an “optimal” use of pens by putting animals from different sources all together in order to avoid having to later clean many dirty pens.

Male sex was a variable that negatively affected indicators of welfare of the calves during penning, with non-castrated males increasing the negative welfare index. Dominance relations among young bulls are unstable [29]. Repeated regroupings challenge the establishment and the maintenance of preferential social and dominance relations and can result in a high level of aggression. This is intensified if space allowances are low and the stocking densities within rest places are high [26]. Fighting was observed in markets between unfamiliar young bulls [11]. The high proportion of mounting among young male cattle is a known issue by markets managers. In some markets, stockpersons put sawdust only in pens designated for male cattle; the high rate of mounting between animals on concrete floor leads to later hoof problems and is appreciated by those involved in this marketing chain (market staff, farmers, and veterinarians).

## 5. Conclusions

In conclusion, the variables associated with the presentation of adverse behaviors in calves related to movement through auction markets (represented by the movement index) were the size of the group handled, the proportion of negative tactile interactions by the handlers, noisy, inappropriate driving, the presence of slippery floor, and inappropriate lighting disrupting the path of the animals. Group size and negative tactile interactions were both predictive variables for the other behaviors index. During penning, not mixing groups from different sources was associated with an increase in the proportion of behavior indicators of positive welfare (represented by the positive welfare index), and increased stocking density was associated with a decrease in the positive welfare index. Variables associated with a decrease in the proportion of negative behavioral indicators (represented by the negative welfare index) were the number of daily auctioned cattle, the observation number (representing passage of time), and when calves were not mixed with incompatible animals and/or with calves from different sources. Male sex in calves was a variable that was associated with an increase in negative behavioral indicators.

These results show that the main factors associated with poor welfare of calves during marketing in auctions were those linked with poor handling and are consequently avoidable, presenting a real and not so difficult opportunity to improve the welfare conditions of the animals marketed through this channel. A shortage of trained and qualified staff could be one reason for the poor welfare seen; additionally, much of the staff was part time. A lack of continuity is an obstacle for workers receiving appropriate training and weakens any commitment and pride that people might feel toward their position. Another important factor, consideration of which might improve welfare, is the sex of the calves—mixing, if it has to be carried out, should be done with due regard to sex, and handling should be tailored to the sex and the temperament. With appropriate management commitment providing opportunities to their stockpeople to participate in training programs, the knowledge and the skills relevant to livestock handling could be improved.

## Figures and Tables

**Table 1:** Definition of the stages evaluated in auction markets.

Stage	Description
Unloading	From the moment truck doors are opened until the last animal comes off the vehicle.
Grading	From the start of sorting until calves are put in a chute to be marked with paint.
Auction	From calves entering the auction ring until leaving it.
Loading	From the lead animal moving toward the vehicle until the last animal is loaded.
Penning	For the duration of holding time in pens.

**Table 2:** Number of groups of animals observed (total number of calves), minimum, maximum, and mean (SD) value of movement and other events index for the groups of calves during movement at each stage of marketing per season.

Stage	Season	Groups	Movement Index		Other Events Index	
		(Calves)	Min–Max	Mean (SD)	Min–Max	Mean (SD)
Unloading	Spring	101 (1232)	0.00–8.00	0.35 (0.86)	0.00–1.25	0.14 (0.25)
	Winter	100 (1180)	0.00–4.00	0.33 (0.58)	0.00–1.17	0.12 (0.25)
Grading	Spring	58 (744)	0.00–4.00	0.60 (0.69)	0.00–3.33	0.20 (0.55)
	Winter	43 (730)	0.10–2.14	0.53 (0.44)	0.00–0.75	0.08 (0.15)
Auction	Spring	162 (642)	0.00–2.00	0.37 (0.54)	0.00–7.00	0.46 (1.13)
	Winter	199 (881)	0.00–3.00	0.42 (0.64)	0.00–14.00	0.54 (1.47)
Loading	Spring	69 (1073)	0.00–14.00	0.49 (1.73)	0.00–3.00	0.09 (0.39)
	Winter	68 (776)	0.00–8.33	0.61 (1.32)	0.00–0.67	0.02 (0.11)

**Table 3:** Parameter estimate, standard error (SE), and significance for the models of average movement index (MI) and other behaviors index (OI) during movement of calves in the market.

Variable	Parameter Estimate	S.E.	*z*-Ratio	*p*-Value
Movement Index (MI)				
Constant	0.368	0.043	8.637	<0.001
Group size	−0.012	0.003	−4.647	<0.001
Negative tactile interactions	0.164	0.007	23.316	<0.001
Driving appropriately				
No (*n* = 345)	0.103	0.051	2.036	0.042
Yes (*n* = 426)	Reference category			
Floor type				
Slippery (*n* = 90)	0.261	0.076	3.429	0.001
Slip-proof (*n* = 688)	Reference category			
Lighting				
Inappropriate (*n* = 2)	1.242	0.457	2.716	0.007
Appropriate (*n* = 796)	Reference category			
Other Events Index (OI)				
Constant	0.424	0.057	7.411	0.000
Negative tactile interactions	0.024	0.010	2.441	0.015
Group size	−0.016	0.004	−4.481	0.000

**Table 4:** Minimum, maximum, and mean (SD) value of the continuous predictor variables in the model of movement index and other events index.

Variable	Min–Max	Mean (SD)
**Tactile interactions (n° events/calves)**	0.00–86.00	0.65 (3.34)
**Group size (number of animals)**	1–60	10 (9.24)

**Table 5:** Number of valid observations (n), minimum (Min), maximum (Max), and mean (SD) value of the positive welfare index (PWI) and the negative welfare index (NWI) obtained during penning of the calves in the market during spring and winter.

Season	Variable	*n*	Min–Max	Mean (SD)
Spring	PWI	210	0.00–1.43	0.18 (0.25)
	NWI	210	0.00–2.33	0.47 (0.43)
Winter	PWI	203	0.00–1.16	0.13 (0.17)
	NWI	203	0.00–2.00	0.37 (0.43)

**Table 6:** Parameter estimate, standard error (SE), and significance for the models of average positive welfare indicators (PWI) and negative welfare indicators (NWI) obtained during penning of the calves in the market.

Explanatory Variables	Parameter Estimate	S.E.	*z*-Ratio	*p*-Value
PWI				
Constant	0.141	0.019	7.605	<0.001
Mixed sources				
No (*n* = 48)	0.092	0.044	2.080	0.038
Yes (*n* = 355)	Reference category			
NWI				
Constant	0.866	0.156	5.541	<0.001
Daily auctioned cattle	−0.0002	0.0001	−2.483	0.013
Observation number	−0.028	0.008	−3.402	0.001
Mixed incompatible				
No (*n* = 405)	−0.530	0.142	−3.727	<0.001
Yes (*n* = 7)	Reference category			
Mixed sources				
No (*n* = 48)	−0.365	0.078	−4.667	<0.001
Yes (*n* = 355)	Reference category			
Sex				
Males (*n* = 302)	0.455	0.069	6.561	<0.001
Mixed (*n* = 5)	0.013	0.292	0.046	0.963
Females (*n* = 100)	Reference category			

**Table 7:** Minimum, maximum, and mean (SD) value of the continuous predictor variables in the model of positive and negative welfare indicators obtained during penning of the calves in the market.

Variable	Min–Max	Mean (SD)
Stocking density (kg/m^2^)	16.61–427.88	136.3 (80.62)
Cattle auctioned (number of animals)	162–1774	791 (492)
